# Post-Stroke Depression and Cognitive Aging: A Multicenter, Prospective Cohort Study

**DOI:** 10.3390/jpm12030389

**Published:** 2022-03-03

**Authors:** Minyoung Shin, Min Kyun Sohn, Jongmin Lee, Deog Young Kim, Yong-Il Shin, Gyung-Jae Oh, Yang-Soo Lee, Min Cheol Joo, So Young Lee, Min-Keun Song, Junhee Han, Jeonghoon Ahn, Young-Hoon Lee, Won Hyuk Chang, Seyoung Shin, Soo Mi Choi, Seon Kui Lee, Yun-Hee Kim

**Affiliations:** 1 Department of Physical and Rehabilitation Medicine, Center for Prevention and Rehabilitation, Heart Vascular Stroke Institute, Samsung Medical Center, Sungkyunkwan University School of Medicine, Seoul 06351, Korea; shinminyoung@sgcp.ac.kr (M.S.); wh.chang@samsung.com (W.H.C.); htl0706@gmail.com (S.S.); 2 Department of Counseling Psychology, Seoul Graduate School of Counseling Psychology, Seoul 03136, Korea; 3 Department of Rehabilitation Medicine, School of Medicine, Chungnam National University, Daejeon 34134, Korea; mksohn@cnu.ac.kr; 4 Department of Rehabilitation Medicine, Konkuk University School of Medicine, Seoul 05030, Korea; leej@kuh.ac.kr; 5 Department and Research Institute of Rehabilitation Medicine, Yonsei University College of Medicine, Seoul 03722, Korea; kimdy@yuhs.ac; 6 Department of Rehabilitation Medicine, Pusan National University School of Medicine, Pusan National University Yangsan Hospital, Yangsan 46241, Korea; rmshin01@gmail.com; 7 Department of Preventive Medicine, Wonkwang University School of Medicine, Iksan 51538, Korea; pmokj@wku.ac.kr (G.-J.O.); lyh8275@hanmail.net (Y.-H.L.); 8 Department of Rehabilitation Medicine, Kyungpook National University School of Medicine, Kyungpook National University Hospital, Daegu 41566, Korea; leeyangsoo@knu.ac.kr; 9 Department of Rehabilitation Medicine, Wonkwang University School of Medicine, Iksan 51538, Korea; jmc77@hanmail.net; 10 Department of Rehabilitation Medicine, Jeju National University School of Medicine, Jeju 63243, Korea; bluelsy900@hanmail.net; 11 Department of Physical and Rehabilitation Medicine, Chonnam National University Medical School, Kwangju 61186, Korea; drsongmk@daum.net; 12 Department of Statistics, Hallym University, Chunchon 24252, Korea; hanjh@hallym.ac.kr; 13 Department of Health Convergence, Ewha Womans University, Seoul 03760, Korea; ahnjeonghoon@ewha.ac.kr; 14 Division of Chronic Disease Prevention, Center for Disease, Korea Disease Control and Prevention Agency, Cheongju 28159, Korea; choism7334@korea.kr (S.M.C.); byuly74@korea.kr (S.K.L.); 15 Samsung Advanced Institute for Health Sciences & Technology, Sungkyunkwan University, Seoul 03063, Korea

**Keywords:** stroke, post-stroke depression, cognitive decline, dementia, older adults

## Abstract

Background: This study investigated the impact of post-stroke depression (PSD) on cognitive aging in elderly stroke patients. Methods: This study was an interim analysis of the Korean Stroke Cohort for Functioning and Rehabilitation. Among 10,636 patients with first-ever stroke, a total of 3215 patients with normal cognitive function three months post-stroke were included in the analysis. PSD was defined using the Korean Geriatric Depression Scale Short Form (K-GDS-SF) at three months. Cognitive aging was defined as a decline in the Korean version of the Mini-Mental Status Examination (K-MMSE) score to less than the second percentile. Results: The hazard ratio (HR) of PSD for cognitive decline was 2.16 (95% CI, 1.34–3.50, *p* < 0.01) in the older group (age ≥65 years), and 1.02 (95% CI, 0.50–2.07, n.s.) in the younger group (age <65 years). When the older group was divided by sex, the HR was 2.50 (95% CI, 1.26–4.96, *p* < 0.01) in male patients and 1.80 (95% CI, 0.93–3.51, n.s.) in female patients. However, women showed a higher incidence of cognitive decline in both the PSD and no PSD groups. Among K-GDS-SF factors, “Negative judgment about the past, present, and future” increased the HR of PSD in older male patients. Conclusions: Early PSD increased the HR for cognitive decline in older stroke patients, mainly in males. Specifically, older male patients with negative thinking were at increased risk of cognitive decline. The findings also suggest that older women may be at risk for cognitive decline. Therefore, preventive interventions for cognitive decline should be tailored differently for men and women.

## 1. Introduction

Geriatric depression is associated with abnormal cognitive aging, including cognitive dysfunction and impairment, as well as dementia [1,2]. The mechanism underlying this process has not been established, but studies have reported relationships between geriatric depression and brain changes, including vascular disease, alterations in the cortisol-hippocampal pathway, increased deposition of amyloid-β plaque formation, inflammatory changes, and decreased levels of nerve growth factors [3]. This pathway is bidirectional, as depression makes the brain more vulnerable to dementia pathology and also can be a sign of underlying brain disease.

The ‘vascular depression’ hypothesis defines geriatric depression in the framework of cerebrovascular disease, proposing that geriatric depression may be influenced by disruption of brain circuits related to mood regulation and cognition [4,5]. Greater white matter hyperintensity (WMH) burden, one of the pathophysiologies of vascular depression, is related to cognitive processing speed, executive function, and memory deficits [4]. Thus, it is not surprising that vascular depression increases the risk of cognitive impairment. Vascular cognitive impairment (VCI) is an umbrella term referring to a wide range of cognitive disorders stemming from cerebrovascular disease [6]. A recent review suggested biomarkers for VCI, including serum concentration of specific proteins, cerebrospinal fluid abnormalities, structural brain changes, and histological changes [6]. These proposed biomarkers will play an important role in identifying, diagnosing, preventing, and treating VCI.

Post-stroke depression (PSD) is a relatively common psychological complication in elderly stroke patients, with most PSD occurring within the first three months after stroke [7]. PSD develops in one-third of stroke patients, is related to post-stroke dementia, and negatively influences recovery and treatment adherence [8,9]; consequently, patients with PSD have a poor prognosis compared to patients without PSD [10]. PSD differs conceptually from vascular depression in that PSD occurs after a clinically apparent stroke. However, PSD and vascular depression are similar with regard to risk factors, clinical features, pathophysiology, treatment, and prognosis [11]. The proposed pathophysiological mechanisms of PSD include glutamate toxicity, abnormal neurotrophic response, lower levels of monoamines, and increased inflammation with hypothalamic–pituitary–adrenal axis dysregulation [12]. PSD occurs after the occurrence of overt brain lesions, and thus disruption of normal brain function appears to affect mood changes.

Psychological distress related to symptoms and lack of social support contributes to PSD development [13]. Considering that geriatric depression and brain pathology interact to accelerate abnormal cognitive aging, PSD, which is affected by bio-psycho-social factors, may also increase the risk of cognitive aging and dementia. In particular, the long-term effects of PSD in older patients should be investigated further, since the aging brain is vulnerable to stroke, lacks resilience, and PSD involves a chronic course. Therefore, even if there is no post-stroke cognitive impairment in the early phase, a history of PSD may increase the long-term risk of dementia or cognitive decline. Since the initial identification of PSD as a major psychological complication of cerebrovascular disease, studies have focused on the diagnosis, prevalence, mortality, treatment and prevention of PSD; risk factors for PSD; and the role of PSD on physical or cognitive recovery [13]. No studies have examined whether the history of PSD affects cognitive aging in stroke patients.

Dementia is an irreversible syndrome without effective treatments, and therefore prevention is the best approach to avoid the adverse effects of dementia pathology. If depression is a sign of underlying brain disease, therapeutic interventions should be initiated early. Some treatments have proven effective against PSD, including medication and cognitive behavior therapy (CBT) [14,15,16]. Regular exercise, social activity, and diet also can lessen symptoms in patients with geriatric depression [17]. Thus, early detection and therapeutic interventions for depression in old age can lower the incidence of dementia.

The purpose of this study was to investigate the effects of PSD on cognitive aging. We hypothesized that PSD increases the risk of cognitive decline in older adult patients, as does geriatric depression. We analyzed a Korean Stroke Cohort for Functioning and Rehabilitation (KOSCO) data and estimated the hazard ratio (HR) of PSD for cognitive decline in older and younger adult patients who had normal cognitive function in the early phase of stroke. In addition, we investigated whether these effects varied by sex, because women are susceptible to depression and have a higher incidence of PSD [18].

## 2. Materials and Methods

### 2.1. Study Design and Participants

This study was an interim analysis of the KOSCO, a 10-year longitudinal, multicenter, prospective cohort study of acute first-ever stroke patients admitted to hospitals in nine regional districts of Korea. The KOSCO was designed to investigate the residual disabilities, activity limitations, and quality of life issues that arise in patients after first-ever stroke. A total of 10,636 patients aged 19 to 100 years with a first-ever stroke from August 2012 to May 2015 were recruited. The five-year follow-up was completed by August 2020. The sample retention rate was 57% at the five-year follow-up. The criteria for inclusion were as follows: (1) first acute stroke (ischemic stroke or intracerebral hemorrhage) with a corresponding lesion or evidence of acute arterial occlusion on computed tomography or magnetic resonance imaging; (2) age ≥ 19 years at stroke onset; and (3) onset of stroke within seven days before inclusion. Patients were excluded if they had a transient ischemic attack, history of stroke, traumatic intracerebral hemorrhage, or were not Korean. Written informed consent was obtained from all patients, or from their legally authorized representative prior to inclusion in the study if the patient was unable to provide informed consent. The ethics committee of each participating hospital approved the study protocol (2012-06-016, 4-2012-0341, 1180-01-700, 2012-06-011, CNUH-2012-127, 05-2012-057, 2013-03-029, 1515, 2013-02-001) A detailed protocol for this study has been published elsewhere [19]. This study protocol was registered on Clinical Trial Registration (NCT03402451). The study follows the Strengthening the Reporting of Observational Studies in Epidemiology guidelines [20].

### 2.2. Measures

The Korean version of the Mini-Mental State Examination (K-MMSE) [21] was used to assess general cognitive function, with scores for orientation, attention and concentration, memory, language, and visuo-constructional function. The total possible score is 30 points. Cognitive aging was defined as a decline from normal to cognitive impairment based on the K-MMSE. Based on age- and education-adjusted norms, normal cognitive function was defined as a score equal to or greater than the 16th percentile, and cognitive impairment was defined as a score less than the second percentile, corresponding to dementia on the K-MMSE [22].

The Korean version of the Geriatric Depression Scale Short Form (K-GDS-SF) [23] was used to assess the presence of depression in participants. This tool consists of 15 questions about depressive symptoms related to negative thoughts about the self and environment, negative feelings, and cognitive dysfunction. PSD was defined as a score of eight or more on the K-GDS-SF at three months [23].

### 2.3. Procedure

This study analyzed data from nine waves (3 months, 6 months, 12 months, 18 months, 24 months, 30 months, 36 months, 48 months, and 60 months) of the KOSCO study. Among the 10,636 patients registered in the KOSCO study, 7858 consented to long-term follow-up. All patients underwent comprehensive surveys and assessments, including motor function, language skills, mood, and cognitive function testing, seven days after stroke onset as a baseline. Patients underwent the same assessments at every follow-up. We included patients who met the following criteria: (1) normal cognitive function assessed by the K-MMSE at three months, and (2) an available score for the K-GDS-SF at three months. Stroke-related cognitive impairment recovers rapidly early after stroke and undergoes a stable maintenance phase [24]. A previous study that analyzed KOSCO data found similar recovery patterns [25]; cognitive impairment recovered rapidly up to the first three months, after which cognitive function remained stable or decreased slightly. Based on these previous studies, we screened patients using cognitive function at three months. Among 7858 patients, 3778 with normal cognitive function at three months were screened. Among these 3778 patients, 552 with no depression score at three months and 11 with premorbid medical conditions that could affect cognitive performance were excluded. A total of 3215 patients was included in the final analysis. In the final patient group, 3215, 2797, 2649, 2583, 2569, 2500, 2492, 2445, and 2347 patients were followed at 3 months, 6 months, 12 months, 18 months, 24 months, and 30 months, 36 months, 48 months, and 60 months, respectively. Each patient attended two to nine follow-up visits; some skipped one or more follow-up sessions. Cases lost to or with missing follow-up visits were coded as censored cases. Among the 3215 patients, 418, 333, 210, 144, 129, 87, 81, and 80 patients were censored at 6 months, 12 months, 18 months, 24 months, and 30 months, 36 months, 48 months, and 60 months, respectively.

**Figure 1:**
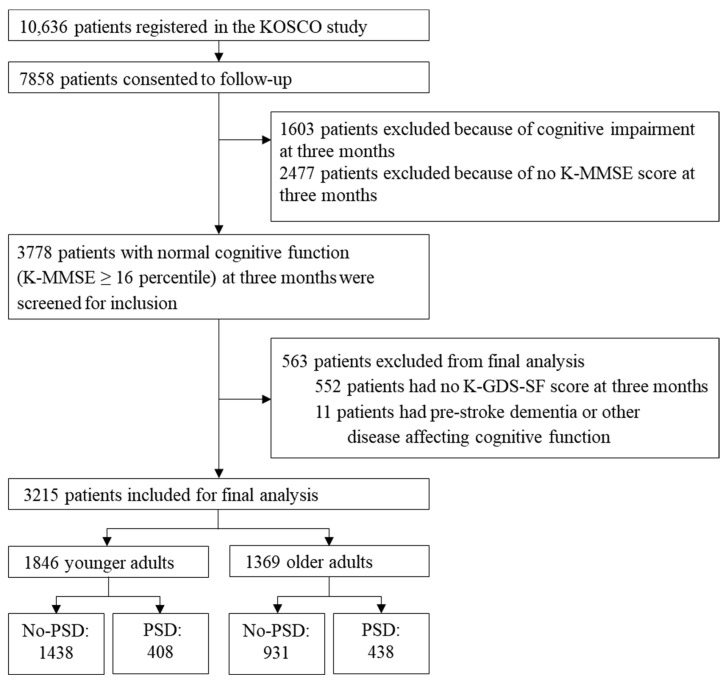
Flow chart of participant enrollment. KOSCO, Korean Stroke Cohort for Functioning and Rehabilitation; K-MMSE, Korean version of the Mini-Mental State Examination; K-GDS-SF, Korean Geriatric Depression Scale Short Form; PSD, post-stroke depression.

Patients were classified into two groups based on depression at three months. Of 3215 patients, 846 had depression (PSD group) and 2369 were not depressed (No-PSD group). We performed survival analysis on younger adult patients (age < 65 years, *n* = 1846) and older adult patients (age ≥ 65 years, *n* = 1369). Among the younger patients, 408 (265 males, 143 females) were in the PSD group and 1438 (1000 males, 438 females) were in the No-PSD group. The prevalence of PSD was 20.9 in males and 24.6 in females. Among older patients, 438 (224 males, 214 females) were in the PSD group and 931 (580 males, 351 females) were in the No-PSD group. The prevalence of PSD was 27.9 in males and 37.9 in females (Figure 1).

### 2.4. Statistical Analysis

Descriptive data from the groups were compared using t-tests or χ² tests. A multivariate Cox proportional hazard model was used to examine the risk of PSD history on cognitive decline adjusted for time-invariant covariates of age, sex, education, and initial K-MMSE score. The HR was obtained with an event defined as a cognitive decline from normal to cognitive impairment. The time to event was calculated from three months after stroke to the month of the event.

We performed additional analysis because MMSE scores fluctuated for some cases across waves even after stroke recurrence was excluded. Situational factors, including the testing environment, tester characteristics, and patient condition at each follow-up session, can affect cognitive performance. Finally, we reviewed the cognitive scores of patients classified as exhibiting cognitive decline. Even if a patient exhibited cognitive impairment at any point in follow-up, if the patient showed normal cognitive performance at their last follow-up evaluation, their case was regarded as remaining normal until that point. IBM SPSS Statistics software version 26.0 [26] was used for all statistical analyses.

## 3. Results

Patient characteristics of the entire study group and the PSD and No-PSD subgroups are presented in Table 1. Among older patients, those with PSD were older than the No-PSD patients. There were more women and more under-educated patients in the PSD group compared with the No-PSD group. Initial K-MMSE scores were lower in PSD patients than No-PSD patients. Among the younger patients, PSD patients were older and less educated than No-PSD patients. Initial K-MMSE scores were lower in PSD patients than No-PSD patients. Initial NIHSS scores, stroke type, and risk factors did not vary between the PSD and the No-PSD patients in both age groups. Thus, age, sex, education, and initial K-MMSE score were controlled in the Cox proportional hazard model in both age groups.

In older patients, cognitive decline was observed in 37 of 931 No-PSD patients (4.0%) and 38 of 438 PSD patients (8.7%). Among these 75 patients, 16, 9, 10, 13, 11, 5, 8, and 3 patients were classified with cognitive decline at 6, 12, 18, 24, 30, 36, 48, and 60 months, respectively. Over five years, 493 of the 931 No-PSD patients (53.0%) and 262 of the 438 PSD patients (59.8%) were censored cases. Among younger patients, cognitive decline was observed in 31 of the 1438 No-PSD patients (2.1%) and 11 of the 408 PSD patients (2.7%). Among these 42 patients, 10, 5, 5, 5, 2, 5, 5, and 5 were classified as having cognitive decline at 6, 12, 18, 24, 30, 36, 48, and 60 months, respectively. Over five years, 701 of the 1438 No-PSD patients (48.7%) and 210 of the 408 PSD patients (51.5%) were censored cases (Table 2). As stroke recurrence can affect cognitive decline, we reviewed interview data of the 75 older and 42 younger patients classified as cognitive decliners, and we excluded patients whose cognitive decline was associated with stroke recurrence. Four cases, including three older and one younger patient, were excluded from the analysis (Table S1) because of stroke recurrence. Therefore, HR was obtained for the final 3211 patients.

**Figure 2:**
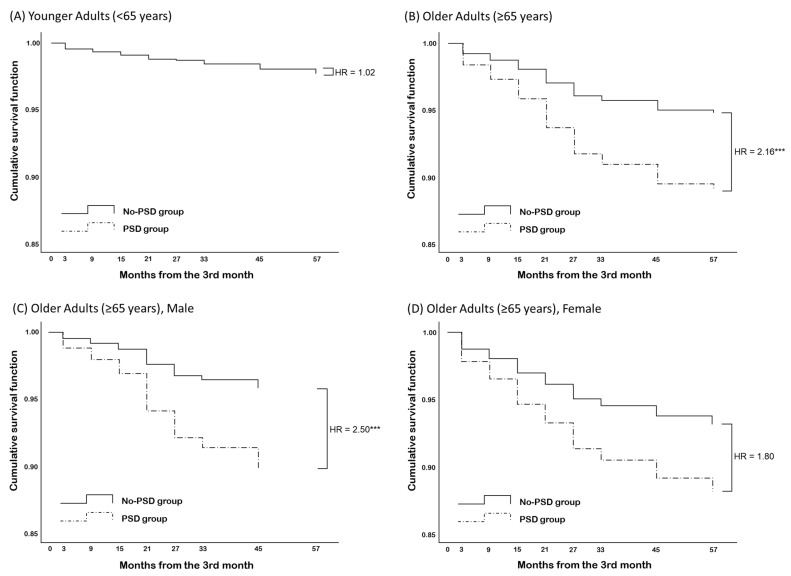
Cumulative survival rates. (**A**) Younger adult patients. (**B**) Older adult patients. (**C**) Older adult patients, male. (**D**) Older adult patients, female. PSD, post-stroke depression; HR, hazard ratio. *** *p* < 0.001.

The proportional hazard assumption was valid for all variables. The log–log survival curves for the PSD and No-PSD groups were approximately parallel, and the parallelism of categories for all other covariates was confirmed. Table 3 and Figure 2 show the results of the Cox proportional hazard models. In older adult patients, the HR of PSD for cognitive decline was 2.16 (95% CI, 1.34–3.50, *p* < 0.01). Limited education (HR = 0.59, 95% CI, 0.33–1.06, n.s.), female sex (HR = 1.35, 95% CI, 0.81–2.26, n.s.), and age (HR = 1.00, 95% CI, 0.96–1.05, n.s.) were not significant. The initial K-MMSE score was negatively related to HR (HR = 0.88, 95% CI, 0.81–0.95, *p* < 0.01). When further calculated by sex, the HR of PSD was 2.50 (95% CI, 1.26–4.96, *p* < 0.01) in male patients and 1.80 (95% CI, 0.93–3.51, n.s.) in female patients (Table S2). The HR was significant only in male patients. We investigated symptoms of depression associated with cognitive decline in each sex; “Negative judgment about the past, present, and future” was significant in male patients (HR = 1.34, 95% CI, 1.09–1.65, *p* < 0.01), but no symptom was significant in female patients (Table 4).

In younger adult patients, the HR of PSD for cognitive decline was not significant (HR = 1.02, 95% CI, 0.50–2.07, n.s.). The control variables were not significant, but the initial K-MMSE score was negatively related to HR (HR = 0.73, 95% CI, 0.61–0.88, *p* < 0.001). When further calculated by sex, the HR of PSD was not significant in either sex (Table S3).

Finally, we performed further analysis to control for fluctuation of cognitive scores across waves. When we reclassified cases considering their last follow-up evaluation, the classification changed from cognitive decline to normal in 16 of 72 older adult patients and 22 of 41 younger adult patients. The results were similar to the original analysis. The HR of PSD for the cognitive decline was significant (HR = 1.94, 95% CI, 1.13–3.35, *p* < 0.05) in the older group but not in the younger group (HR = 1.67, 95% CI, 0.70–4.13, n.s.) (Table S4).

## 4. Discussion

We investigated the effect of PSD history on cognitive aging. Our study demonstrated that PSD increased the risk of cognitive decline in older adult patients, mainly males, over the five years following a stroke, but not in younger adult patients. Furthermore, among the symptoms of depression, “Negative judgment about the past, present, and future” significantly increased risk in older male patients, but no symptom was significant in older female patients.

The present study showed that PSD, like geriatric depression [1], is a risk factor for abnormal cognitive aging in older adult patients without cognitive impairment at baseline. Few studies have investigated cognitive aging in patients with a history of PSD. One study reported that psychiatric symptoms (not limited to depression) increased the risk of cognitive decline following stroke [27]. However, 82% of these patients were diagnosed with vascular dementia or post-stroke mild cognitive impairment at baseline, in contrast to the present study. Our findings suggest that active therapeutic interventions for depression are needed to prevent dementia, even if cognitive dysfunction does not immediately present after stroke.

The impact of PSD on cognitive decline was not significant in younger patients. This result remained the same when HR was estimated by sex. No previous study had compared the long-term effect of PSD between younger and older patients. The aging brain has a greater vascular burden [28] and lower brain plasticity than the young brain [29] and may show low resilience after damage. In addition, PSD can interact with psychosocial factors, limiting daily functioning, social interactions, and mobility [13]. These factors increase the risk for dementia because they reduce stimulation of the brain. Our results also demonstrated that PSD is more hazardous in elderly patients.

Additional subgroup analysis by sex in older patients suggested that the impact of PSD on cognitive decline was greater in male stroke patients, showing a significant HR of PSD for cognitive decline. The HR of PSD was not significant in female patients. In addition, further analysis of depression symptoms showed that “Negative judgment about the past, present, and future” significantly increased risk in male patients. Negative feelings about their situation in the early stage of treatment can lead patients to be skeptical of treatment, reduce compliance, and limit social interaction and activities. These factors can be related to dementia risk and are associated with cognitive decline in male patients. No depression symptom was significantly related to cognitive decline in female patients.

In our analysis, PSD was more prevalent in females than males, which was consistent with previous studies [12,18], and depressive symptoms were more severe in female patients in the No-PSD group (Table S5). These results suggest that PSD is more common in women, and female patients experience subthreshold depression more frequently than male patients. Therefore, categorizing PSD based on a specific cut-off score may not provide useful information to predict cognitive decline in women. Our data also showed that older female patients had a higher rate of cognitive decline than older male patients in both the PSD group (9.4% in females vs. 8.0% in males) and the No-PSD group (4.9% in females vs. 2.9% in males). In addition, although HR was not significant, the risk for cognitive decline was higher in females than males (HR = 1.35). Thus, female sex appears to be a risk factor for cognitive decline. In this study, women had lower initial K-MMSE scores than men (Table S5), and initial K-MMSE score was a significant predictor for cognitive decline in older female patients (Table S2). Thus, low cognitive function after stroke may explain the high incidence of cognitive decline in women.

Stroke recurrence or other situational factors can affect cognitive decline [30]. In the original analysis, cases with recurrence-related cognitive decline were excluded from analysis. However, all but four recurrent cases maintained normal cognitive function or showed only mild decline. Therefore, recurrence does not seem to be the main cause of cognitive decline in our dataset. There were some cases where cognitive scores fluctuated across waves, which suggested that situational factors might affect cognitive function. We reviewed fluctuation data, reclassified cases considering their last follow-up evaluation, and performed further analysis. The results remained the same as the original analysis, supporting our main results. Fluctuation was more prevalent in younger patients than in older patients. Therefore, there must be other causes of cognitive decline in younger patients because cognitive fluctuation, a reversion from cognitive impairment to normal, is not a typical pattern of cognitive aging. This should be investigated in further studies.

Dementia is irreversible once it develops and there currently are no effective treatments. Therefore, prevention is the most effective measure. Previous studies have shown that antidepressant treatment initiated in the early stage of stroke might prevent PSD development and positively affect long-term functional recovery [14,15]; CBT combined with antidepressants also showed efficacy for PSD [16]. However, there are no practical guidelines for the treatment of PSD [31]. Our findings provide support for early detection and treatment of PSD.

This study had several strengths. First, it is the first and only large-scale study to examine the impact of PSD on cognitive aging over five years. The results of this study highlight the need for early intervention in depression, as PSD can increase the risk of abnormal cognitive aging in older male stroke patients. Second, we studied patients with normal cognitive function after stroke. The findings suggest that, even if cognitive impairment is absent or has recovered to normal after stroke, the risk of abnormal cognitive aging remains in the presence of PSD. Finally, we attempted to control for cognitive fluctuation across waves.

This study also had several limitations. First, since PSD was defined based on a self-rated depression scale, there was a limit to the accuracy of diagnosis. Nevertheless, the results of the study highlight that even a self-rated depression scale can predict an increased risk of cognitive decline over five years. Second, premorbid depression and a family history of depression might affect PSD, but we were unable to analyze these factors as these data were unavailable. Third, we set the criterion for cognitive decline as less than the second percentile, corresponding to dementia, and excluded data if the decline was related to stroke recurrence. However, since cognitive decline was not a clinical diagnosis, we cannot exclude the possibility that cognitive decline was due to causes other than dementia. Fourth, the K-MMSE is not as sensitive to cognitive decline as are comprehensive neuropsychological batteries. Thus, the actual number of cases experiencing cognitive decline is likely greater than estimated in this study. Finally, recent studies have focused on neural circuits or functional connectivity changes to understand emotional regulation, emotional processing, and cognitive deficits [32,33]; such measures are important for understanding depression and cognitive impairment after stroke, since stroke damages brain networks. Future studies should address whether the disruption of brain circuits associated with PSD predicts cognitive decline.

## 5. Conclusions

In conclusion, a history of early PSD increases the risk of cognitive decline in older adult patients, mainly males, over the five years following a stroke. This study also suggests that older women may be at risk for cognitive decline. Therefore, preventive interventions for cognitive decline after stroke should be tailored differently for men and women. In addition, medical staff and caregivers should closely monitor patients for chronic negative thinking, because this symptom is closely associated with cognitive decline in older male patients.

## Figures and Tables

**Table 1:** Baseline characteristics of the subjects.

	Younger Adults (<65 Years), *n* = 1846			Older Adults (≥65 Years), *n* = 1369		
Variables	No-PSD	PSD	t/x^2^	No-PSD	PSD	t/x^2^
*n*	1438	408	N/A	931	438	N/A
Age, years	51.83 (9.0)	52.87 (8.9)	−2.05 *	72.99 (5.6)	73.91 (5.9)	−2.78 **
Female sex	438 (30.5)	143 (35.0)	3.11	351(37.7)	214 (48.9)	15.30 ***
Limited education (<9 years)	131 (9.1)	56 (13.7)	7.44 **	387 (41.6)	233 (53.2)	16.26 ***
Initial NIHSS	4.00 (5.4)	3.58 (4.9)	1.45	4.05 (5.5)	3.47 (5.0)	1.96
Ischemic type	1082 (75.2)	295 (72.3)	1.45	837 (89.9)	392 (89.5)	0.05
Hypertension	634 (44.1)	170 (41.7)	0.76	614 (66.0)	284 (64.8)	0.16
Diabetes mellitus	250 (17.4)	81 (19.9)	1.32	266 (28.6)	121 (27.6)	0.13
Coronary heart disease	65 (4.5)	10 (2.5)	3.49	91 (9.8)	38 (8.7)	0.42
Atrial fibrillation	65 (4.5)	25 (6.1)	1.77	126 (13.5)	55 (12.6)	0.25
Left ventricular hypertrophy	17 (1.2)	4 (1.0)	0.12	7 (0.8)	6 (1.4)	1.20
Peripheral artery disease	5 (0.3)	1 (0.2)	0.10	9 (1.0)	4 (0.9)	0.01
Hyperlipidemia	210 (14.6)	58 (14.2)	0.04	154 (16.5)	69 (15.8)	0.14
Low cholesterol	39 (2.7)	17 (4.2)	2.29	38 (4.1)	15 (3.4)	0.35
Unruptured intracranial aneurysm	21 (1.5)	7 (1.7)	0.14	9 (1.0)	6 (1.4)	0.45
Arteriovenous malformation	5 (0.3)	2 (0.5)	0.17	3 (0.3)	1 (0.2)	0.09
Moyamoya disease	14 (1.0)	4 (1.0)	0.00	2 (0.2)	1 (0.2)	0.00
Obesity	214 (14.9)	50 (12.3)	1.79	118 (12.7)	54 (12.3)	0.03
Smoking	699 (48.6)	202 (49.5)	0.10	296 (31.8)	138 (31.5)	0.01
Alcohol consumption	775 (53.9)	207 (50.7)	1.27	317 (34.0)	132 (30.1)	2.07
K-GDS-SF at 3 months	3.03 (2.0)	10.65 (2.2)	−63.92 ***	3.50 (2.1)	10.75 (2.3)	−56.07 ***
K-MMSE at 3 months	28.87 (1.4)	28.44 (1.5)	5.12 ***	26.90 (2.8)	25.29 (3.6)	8.33 ***

Data are shown as *n* (%) or mean (standard deviation). PSD, post-stroke depression; NIHSS, National Institutes of Health Stroke Scale; K-GDS-SF, Korean Geriatric Depression Scale Short Form; K-MMSE, Korean version of the Mini-Mental State Examination. * *p* < 0.05, ** *p* < 0.01, *** *p* < 0.001.

**Table 2:** Number of normal, cognitive decline, and censored patients at each time point.

		Younger Adults (<65 Years), *n* = 1846				Older Adults (≥65 Years), *n* = 1369			
Months	Groups	Normal	Cognitive Decline	Censored	Total	Normal	Cognitive Decline	Censored	Total
3	No-PSD	1438 (100)	0 (0)	0 (0)	1438 (100)	931 (100)	0 (0)	0 (0)	931 (100)
	PSD	408 (100)	0 (0)	0 (0)	408 (100)	438 (100)	0 (0)	0 (0)	438 (100)
	Total	1846 (100)	0 (0)	0 (0)	1846 (100)	1369 (100)	0 (0)	0 (0)	1369 (100)
6	No-PSD	1250 (86.9)	5 (0.3)	183 (12.7)	1438 (100)	802 (86.1)	7 (0.8)	122 (13.1)	931 (100)
	PSD	347 (85.0)	5 (1.2)	56 (13.7)	408 (100)	363 (82.9)	9 (2.1)	66 (15.1)	438 (100)
	Total	1597 (86.5)	10 (0.5)	239 (12.9)	1846 (100)	1165 (85.1)	16 (1.2)	188 (13.7)	1369 (100)
12	No-PSD	1099 (87.9)	5 (0.4)	146 (11.7)	1250 (100)	690 (86.0)	2 (0.2)	110 (13.7)	802 (100)
	PSD	307 (88.5)	0 (0.0)	40 (11.5)	347 (100)	289 (79.6)	7 (1.9)	67 (18.5)	363 (100)
	Total	1406 (88.0)	5 (0.3)	186 (11.6)	1597 (100)	979 (84.0)	9 (0.8)	177 (15.2)	1165 (100)
18	No-PSD	993 (90.4)	4 (0.4)	102 (9.3)	1099 (100)	624 (90.4)	4 (0.6)	62 (9.0)	690 (100)
	PSD	269 (87.6)	1 (0.3)	37 (12.1)	307 (100)	242 (83.7)	6 (2.1)	41 (14.2)	289 (100)
	Total	1262 (89.8)	5 (0.4)	139 (9.9)	1406 (100)	866 (88.5)	10 (1.0)	103 (10.5)	979 (100)
24	No-PSD	902 (90.8)	4 (0.4)	87 (8.8)	993 (100)	579 (92.8)	7 (1.1)	38 (6.1)	624 (100)
	PSD	249 (92.6)	1 (0.4)	19 (7.1)	269 (100)	214 (88.4)	6 (2.5)	22 (9.1)	242 (100)
	Total	1151 (91.2)	5 (0.4)	106 (8.4)	1262 (100)	793 (91.6)	13 (1.5)	60 (6.9)	866 (100)
30	No-PSD	838 (92.9)	2 (0.2)	62 (6.9)	902 (100)	529 (91.4)	9 (1.6)	41 (7.1)	579 (100)
	PSD	222 (89.2)	0 (0.0)	27 (10.8)	249 (100)	193 (90.2)	2 (0.9)	19 (8.9)	214 (100)
	Total	1060 (92.1)	2 (0.2)	89 (7.7)	1151 (100)	722 (91.0)	11 (1.4)	60 (7.6)	793 (100)
36	No-PSD	783 (93.4)	4 (0.5)	51 (6.1)	838 (100)	494 (93.4)	3 (0.6)	32 (6.0)	529 (100)
	PSD	211 (95.0)	1 (0.5)	10 (4.5)	222 (100)	175 (90.7)	2 (1.0)	16 (8.3)	193 (100)
	Total	994 (93.8)	5 (0.5)	61 (5.8)	1060 (100)	669 (92.7)	5 (0.7)	48 (6.6)	722 (100)
48	No-PSD	751 (95.9)	3 (0.4)	29 (3.7)	783 (100)	445 (90.1)	4 (0.8)	45 (9.1)	494 (100)
	PSD	197 (93.4)	2 (0.9)	12 (5.7)	211 (100)	155 (88.6)	4 (2.3)	16 (9.1)	175 (100)
	Total	948 (95.4)	5 (0.5)	41 (4.1)	994 (100)	600 (89.7)	8 (1.2)	61 (9.1)	669 (100)
60	No-PSD	706 (94.0)	4 (0.5)	41 (5.5)	751 (100)	401(90.1)	1(0.2)	43(9.7)	445(100)
	PSD	187 (94.9)	1 (0.5)	9 (4.6)	197 (100)	138(89.0)	2(1.3)	15(9.7)	155(100)
	Total	893 (94.2)	5 (0.5)	50 (5.3)	948 (100)	539(89.8)	3(0.5)	58(9.7)	600(100)
Total	No-PSD	706 (49.1)	31 (2.1)	701 (48.7)	1438 (100)	401 (43.1)	37 (4.0)	493 (53.0)	931 (100)
	PSD	187 (45.8)	11 (2.7)	210 (51.5)	408 (100)	138 (31.5)	38 (8.7)	262 (59.8)	438 (100)
	Total	893 (48.4)	42 (2.3)	911 (49.3)	1846 (100)	539 (39.4)	75 (5.5)	755 (55.1)	1369 (100)

Data are shown as *n* (%). PSD, post-stroke depression.

**Table 3:** The hazard ratios of PSD adjusted for background variables by age group.

	Younger Adults (<65 Years), *n* = 1845		Older Adults (≥65 Years), *n* = 1366	
Variables	Estimate (SE)	Hazard Ratio (95% CI)	Estimate (SE)	Hazard Ratio (95% CI)
Age	0.40 (0.03)	1.04 (0.99–1.09)	0.00 (0.02)	1.00 (0.96–1.05)
Limited education (<9 years)	0.72 (0.39)	2.04 (0.95–4.40)	−0.53 (0.30)	0.59 (0.33–1.06)
Female sex	−0.25 (0.34)	0.78 (0.40–1.53)	0.30 (0.26)	1.35 (0.81–2.26)
K-MMSE at 3 months	−0.31 (0.09) ***	0.73 (0.61–0.88)	−0.13 (0.04) **	0.88 (0.81–0.95)
PSD	0.19 (0.36)	1.02 (0.50–2.07)	0.77 (0.25) **	2.16 (1.34–3.50)

K-MMSE, Korean version of the Mini-Mental State Examination; PSD, post-stroke depression; SE, standard error; CI, confidence interval. ** *p* < 0.01, *** *p* < 0.001.

**Table 4:** K-GDS-SF factors associated with higher hazard ratios by sex in older adults.

	Male, *n* = 803		Female, *n* = 563	
Variables	Estimate (SE)	Hazard Ratio (95% CI)	Estimate (SE)	Hazard Ratio (95% CI)
Age	0.04 (0.03)	1.04 (0.97–1.11)	−0.03 (0.03)	0.97 (0.91–1.03)
Limited education (<9 years)	−0.66 (0.44)	0.52 (0.22–1.22)	−0.49 (0.42)	0.61 (0.27–1.39)
K-MMSE at 3 months	−1.15 (0.07) *	0.86 (0.75–1.00)	−0.16 (0.05) *	0.86 (0.77–0.95)
K-GDS-SF, factor 1	0.29 (0.11) **	1.34 (1.09–1.65)	0.05 (0.11)	1.05 (0.86–1.29)
K-GDS-SF, factor 2	0.05 (0.17)	1.05 (0.75–1.47)	0.32 (0.17)	1.37 (0.98–1.92)
K-GDS-SF, factor 3	−0.23 (0.20)	0.80 (0.54–1.17)	−0.33 (0.19)	0.72 (0.49–1.04)

K-MMSE, Korean version of the Mini-Mental State Examination; K-GDS-SF, Korean Geriatric Depression Scale Short Form; SE, standard error; CI, confidence interval; factor 1, Negative judgment about the past, present, and future; factor 2, lowered affect; factor 3, cognitive inefficiency and a lack of motivation. * *p* < 0.05, ** *p* < 0.01.

## Data Availability

The study data cannot be accessed publicly per the internal regulations of the Korean National Institute of Health because KOSCO (Korean Stroke Cohort for Functioning and Rehabilitation) is an ongoing project.

## Supplementary Materials

The following supporting information can be downloaded at: https://www.mdpi.com/article/10.3390/jpm12030389/s1, Table S1: Recurrence vs. recurrence cases among cognitive decliners; Table S2: The hazard ratios of PSD adjusted for background variables by sex in older adult patients; Table S3: The hazard ratios of PSD adjusted for background variables by sex in younger patients; Table S4: The hazard ratios of PSD controlling cognitive fluctuation across waves, adjusted for background variables; Table S5: Comparison of baseline K-GDS-SF and K-MMSE scores by sex in older patients.
