# Supplementary material for: Post-Stroke Depression and Cognitive Aging: A Multicenter, Prospective Cohort Study
Source: J Pers Med. 2022 Mar 3;12(3):389. doi: 10.3390/jpm12030389 (PMC8952332; doi:10.3390/jpm12030389)
Supplement: Supplementary file 1 [file jpm-12-00389-s001.zip › jpm-1597051-supplementary.pdf]

SUPPLEMENTARY MATERIALS

Post-Stroke Depression and Cognitive Aging: A multicenter, prospective cohort study

Minyoung Shin[1, 2], Min Kyun Sohn[3], Jongmin Lee[4], Deog Young Kim[5], Yong-Il Shin[6], Gyung-Jae Oh[7], Yang-Soo Lee[8], Min Cheol Joo[9], So Young Lee[10], Min-Keun Song[11], Junhee Han[12], Jeonghoon Ahn[13], Young-Hoon Lee[7], Won Hyuk Chang[1], Seyoung Shin[1], Soo Mi Choi[14], Seon Kui Lee[14] and Yun-Hee Kim[1,15,*]

[1]Department of Physical and Rehabilitation Medicine, Center for Prevention and Rehabilitation, Heart Vascular Stroke Institute, Samsung Medical Center, Sungkyunkwan University School of Medicine, Seoul, Republic of Korea; [2]Department of Counseling Psychology, Seoul Graduate School of Counseling Psychology, Seoul, Republic of Korea; [3]Department of Rehabilitation Medicine, School of Medicine, Chungnam National University, Daejeon, Republic of Korea; [4]Department of Rehabilitation Medicine, Konkuk University School of Medicine, Seoul, Republic of Korea; [5]Department and Research Institute of Rehabilitation Medicine, Yonsei University College of Medicine, Seoul, Republic of Korea; [6]Department of Rehabilitation Medicine, Pusan National University School of Medicine, Pusan National University Yangsan Hospital, Yangsan, Republic of Korea; [7]Department of Preventive Medicine, Wonkwang University School of Medicine, Iksan, Korea; [8]Department of Rehabilitation Medicine, Kyungpook National University School of Medicine, Kyungpook National University Hospital, Daegu, Republic of Korea; [9]Department of Rehabilitation Medicine, Wonkwang University School of Medicine, Iksan, Republic of Korea; [10]Department of Rehabilitation Medicine, Jeju National University School of Medicine, Jeju, Republic of Korea; [11]Department of Physical and Rehabilitation Medicine, Chonnam National University Medical School, Kwangju, Republic of Korea; [12]Department of Statistics, Hallym University, Chunchon, Republic of Korea; [13]Department of Health Convergence, Ewha Womans University, Seoul, Republic of Korea; [14]Division of Chronic Disease Prevention, Center for Disease, Korea Disease Control and Prevention Agency, Cheongju, Republic of Korea; [15]Department of Health Science and Technology, Department of Medical Device Management and Research, Department of Digital Healthcare, SAIHST, Sungkyunkwan University, Seoul, Republic of Korea

*Correspondence and reprint requests:

Yun-Hee Kim, MD, PhD, Department of Physical and Rehabilitation Medicine, Center for Prevention and Rehabilitation, Heart Vascular Stroke Institute, Samsung Medical Center, Sungkyunkwan University School of Medicine, Department of Health Science and Technology, Department of Medical Device Management and Research, Department of Digital Healthcare, SAIHST, Sungkyunkwan University, 81 Irwon-ro, Gangnam-gu, Seoul, 06351, Republic of Korea

Tel: 82-2-3410-2824

E-mail: yunkim@skku.edu; yun1225.kim@samsung.com

Table S1. Recurrence vs. no recurrence cases among cognitive decliners

Months	Younger adult patients (<65 years), N = 42		Older adult patients (≥65 years), N = 75	
	No recurrence	Recurrence	No recurrence	Recurrence
6	10	0	16	0
12	5	0	9	0
18	5	0	10	0
24	5	0	13	0
30	2	0	11	0
36	4	1	4	1
48	5	0	7	1
60	5	0	2	1
Total	41	1	72	3

Table S2. The hazard ratios of PSD adjusted for background variables by sex in older adult patients

	Male, N = 803		Female, N = 563	
Variables	Estimate (SE)	Hazard Ratio (95% CI)	Estimate (SE)	Hazard Ratio (95% CI)
Age	0.04 (0.03)	1.04 (0.98–1.11)	-0.03 (0.03)	0.97 (0.92–1.03)
Limited education (<9years)	-0.66 (0.44)	0.52 (0.22–1.23)	-0.46 (0.41)	0.63 (0.28–1.42)
K-MMSE at 3mo	-0.13 (0.07)	0.88 (0.76–1.02)	-0.14 (0.05)**	0.87 (0.79–0.96)
PSD	0.92 (0.35)**	2.50 (1.26–4.96)	0.59 (0.34)	1.80 (0.93–3.51)

K-MMSE: Korean version of the Mini-Mental State Examination; PSD: post-stroke depression; SE: standard error; CI: confidence interval. ** p <0.01.

Table S3. The hazard ratios of PSD adjusted for background variables by sex in younger patients

	Male, N = 1265		Female, N = 580	
Variables	Estimate (SE)	Hazard Ratio (95% CI)	Estimate (SE)	Hazard Ratio (95% CI)
Age	0.03 (0.03)	1.03 (0.98–1.09)	0.05 (0.05)	1.05 (0.96–1.15)
Limited education (<9 years)	1.14 (0.45)*	3.12 (1.29–7.54)	-0.23 (0.73)	0.79 (0.19–3.30)
K-MMSE at 3 mo	-0.24 (0.11)*	0.79 (0.63–0.98)	-0.48 (0.17)**	0.62 (0.44–0.87)
PSD	0.24 (0.43)	1.28 (0.55–2.95)	-0.37 (0.67)	0.69 (0.19–2.57)

K-MMSE: Korean version of the Mini-Mental State Examination; PSD: post-stroke depression; SE: standard error; CI: confidence interval. * p <0.05, ** p <0.01.

Table S4. The hazard ratios of PSD controlling cognitive fluctuation across waves, adjusted for background variables

	Younger adult patients (<65 years), N = 1845		Older adult patients (≥65 years), N = 1366	
Variables	Estimate (SE)	Hazard Ratio (95% CI)	Estimate (SE)	Hazard Ratio (95% CI)
Age	0.03 (0.03)	1.03 (0.97–1.10)	0.01 (0.02)	1.01 (0.96–1.06)
Limited education (<9 years)	0.83 (0.53)	2.30 (0.82–6.47)	-0.39 (0.34)	0.68 (0.35–1.31)
Female sex	-0.75 (0.52)	0.47 (0.59–0.97)	0.16 (0.30)	1.17 (0.65–2.10)
K-MMSE at 3 mo	-0.28 (0.13)*	0.76 (0.59–0.97)	-0.14 (0.05)**	0.87 (0.79–0.95)
PSD	0.53 (0.45)	1.67 (0.70–4.13)	0.67 (0.29)*	1.94 (1.13–3.35)

K-MMSE: Korean version of the Mini-Mental State Examination; PSD: post-stroke depression; SE: standard error; CI: confidence interval. * $p < 0.05$, ** $p < 0.01$.

Table S5. Comparison of baseline K-GDS-SF and K-MMSE scores by sex in older patients

	PSD group, N = 438			No-PSD group, N = 931			Total, N = 1269		
Variables	Male	Female	t	Male	Female	t	Male	Female	t
n	224	214	N/A	580	351	N/A	804	565	N/A
K-GDS-SF at 3mo	10.64 (2.27)	10.86 (2.31)	-0.97	3.28 (2.10)	3.87 (2.02)	-4.26***	5.32 (3.94)	6.52 (4.01)	-5.46***
K-MMSE at 3mo	26.13 (2.74)	24.41 (4.09)	5.16***	27.49 (2.26)	25.93 (3.31)	7.79***	27.11 (2.48)	25.35 (3.70)	9.85***

Data are shown as mean (standard deviation). PSD: post-stroke depression; K-GDS-SF: Korean Geriatric Depression Scale Short Form; K-MMSE: Korean version of the Mini-Mental State Examination. *** $p < 0.001$.
